# Supplementary material for: Precision RNAi in Tomato Using Synthetic Trans‐Acting Small Interfering RNAs Derived From Minimal Precursors
Source: Plant Biotechnol J. 2025 Oct 14;24(3):1269–84. doi: 10.1111/pbi.70410 (PMC12946458; doi:10.1111/pbi.70410)
Supplement: Supplementary file 4 — Figure S1: Direct syn‐tasiRNA cloning downstream the SlmiR482b target site (TS) in B/c (BsaI/ccdB)‐ Figure S2: Phasing analysis of 21‐nt reads corresponding to SlSFT and SlSFT. Figure S3: Comparative analysis of gene silencing induced by MIGS and syn‐tasiRNAs in Solanum. Figure S4: Functional analysis of dsRNA treatments against tomato spotted wilt virus (TSWV) in S. Figure S5: Analysis of SlmiR482b and SlmiR6020 presence in Solanum lycopersicum agroinfiltrated. Figure S6: Functional analysis PVX‐based syn‐tasiR‐VIGS silencing of SlSP/SlSP5G and SlCCD8. Table S1: Name, sequence and use of oligonucleotides used in this study. Text S1: Protocol to design and clone syn‐tasiRNAs downstream the 3′D1[+] position in BsaI/ccdBbased (‘B/c’) vectors pENTR‐SlmiR482bTS‐B/c and pMDC32B‐SlmiR482bTS‐B/c. Text S2: Protocol to generate PVX‐based syn‐tasiRNA constructs. Text S3: DNA sequence in FASTA format of all precursors used to express art‐sRNAs in plants. Text S4: DNA sequence of BsaI‐ccdB‐based (B/c) vectors used for direct cloning of syn‐tasiRNAs. [file PBI-24-1269-s001.pdf]

SUPPORTING INFORMATION

Data S1. 21-nt siRNAs from *SlSFT* and *SlLRR1*.

Data S2. P-SAMS designs of art-sRNA sequences.

Data S3. sRNA reads from syn-tasiRNA-expressing tissues.

Figure S1. Direct syn-tasiRNA cloning downstream the SlmiR482b target site (TS) in B/c (*Bsa*I/*ccd*B)-based vectors including a *ccd*B cassette flanked by two *Bsa*I sites.

Figure S2. Phasing analysis of 21-nt reads corresponding to *SlSFT* and *SlSFT*.

Figure S3. Comparative analysis of gene silencing induced by MIGS and syn-tasiRNAs in *Solanum lycopersicum*.

Figure S4. Functional analysis of dsRNA treatments against tomato spotted wilt virus (TSWV) in *S. lycopersicum*.

Figure S5. Analysis of SlmiR482b and SlmiR6020 presence in *Solanum lycopersicum* agroinfiltrated tissues.

Figure S6. Functional analysis PVX-based syn-tasiR-VIGS silencing of *SlSP*/*SlSP5G* and *SlCCD8* silencing in *Solanum lycopersicum*.

Table S1. Name, sequence and use of oligonucleotides used in this study.

Text S1. Protocol to design and clone syn-tasiRNAs downstream the 3'D1[+] position in *Bs*aI/*ccd*B-based ('B/c') vectors *pENTR-SlmiR482bTS-B/c* and *pMDC32B-SlmiR482bTS-B/c*.

Text S2. Protocol to generate PVX-based syn-tasiRNA constructs.

Text S3. DNA sequence in FASTA format of all precursors used to express art-sRNAs in plants.

Text S4. DNA sequence of *Bsa*I-*ccd*B-based (B/c) vectors used for direct cloning of syn-tasiRNAs.

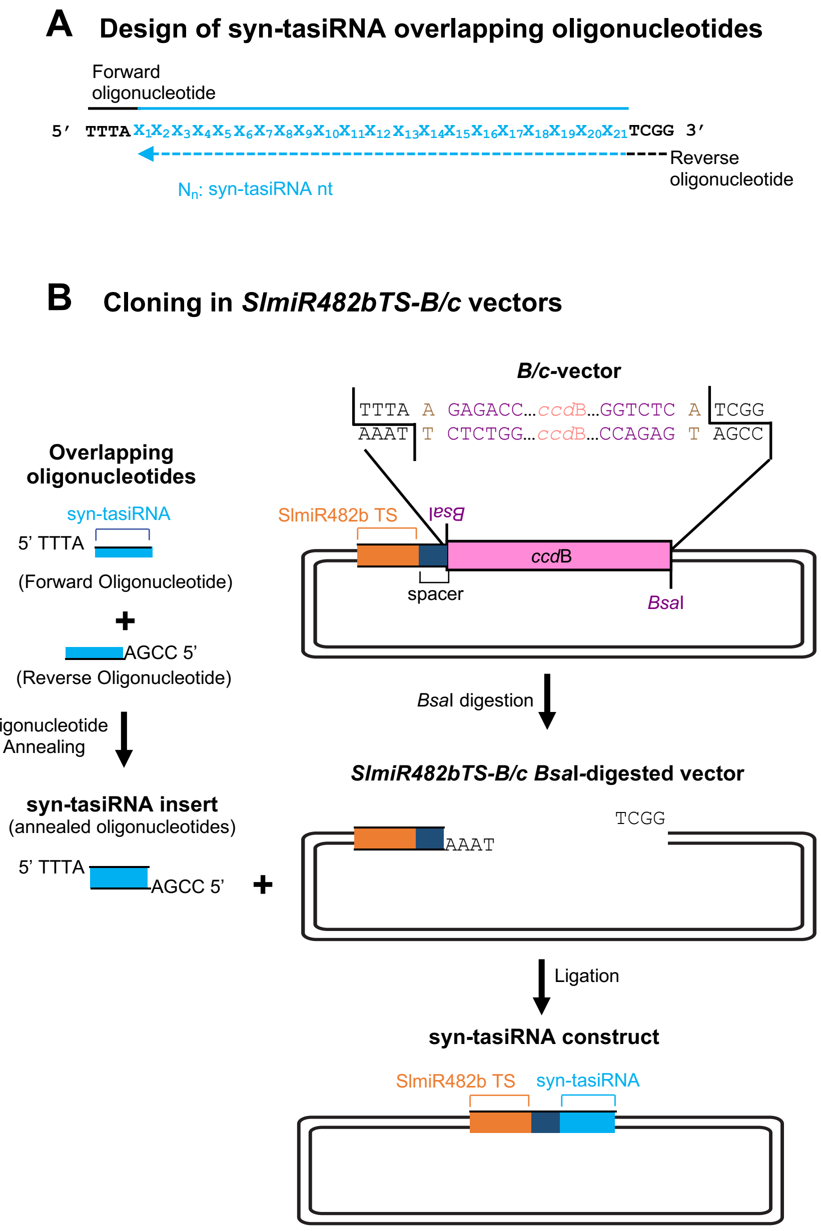

Figure S1. Direct syn-tasiRNA cloning downstream the SlmiR482b target site (TS) in *B/c* (*Bsa*I/*ccd*B)-based vectors including a *ccd*B cassette flanked by two *Bsa*I sites. (**A**) Design of two overlapping oligonucleotides for syn-tasiRNA cloning. Sequence covered by the forward and reverse oligonucleotides are represented with continuous or dotted lines, respectively. Nucleotides of the syn-tasiRNA sequence are in blue, and oligonucleotide 5' overhangs are in black and bold. (**B**) Diagram of the steps for syn-tasiRNA cloning in *SlmiR482bTS-B/c* vectors. The syn-tasiRNA insert obtained after annealing the two overlapping oligonucleotides has 5' TTTA and 5'-CCGA overhangs and is directly inserted into the *Bsa*I-linearized *SlmiR482bTS-B/c*-based vectors. Nucleotides of the *Bsa*I sites and arbitrary nucleotides used as spacers between the *Bsa*I recognition site and the *AtTAS1c* sequence are in purple and light brown, respectively. Nucleotides of the SlmiR482b TS are in orange. The *AtTAS1c*-derived spacer and the syn-tasiRNA sequences are in dark and light blue, respectively.

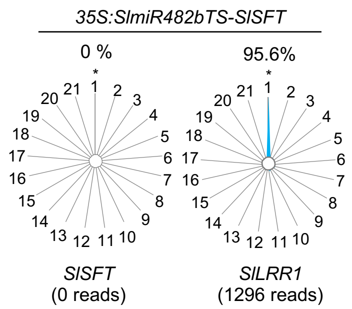

Figure S2. Phasing analysis of amiRNA target *SlSFT* mRNA-derived 21 nucleotide small RNAs. Radar plots show proportions of 21-nucleotide reads corresponding to each of the 21 registers from *SlFT*, with position 1 designated as immediately after the amiRNA guided cleavage site. Control plot for tasiRNA-generating *SlLRR1* is shown. The percentage of 21-nucleotide reads corresponding to phasing register 1 is indicated.

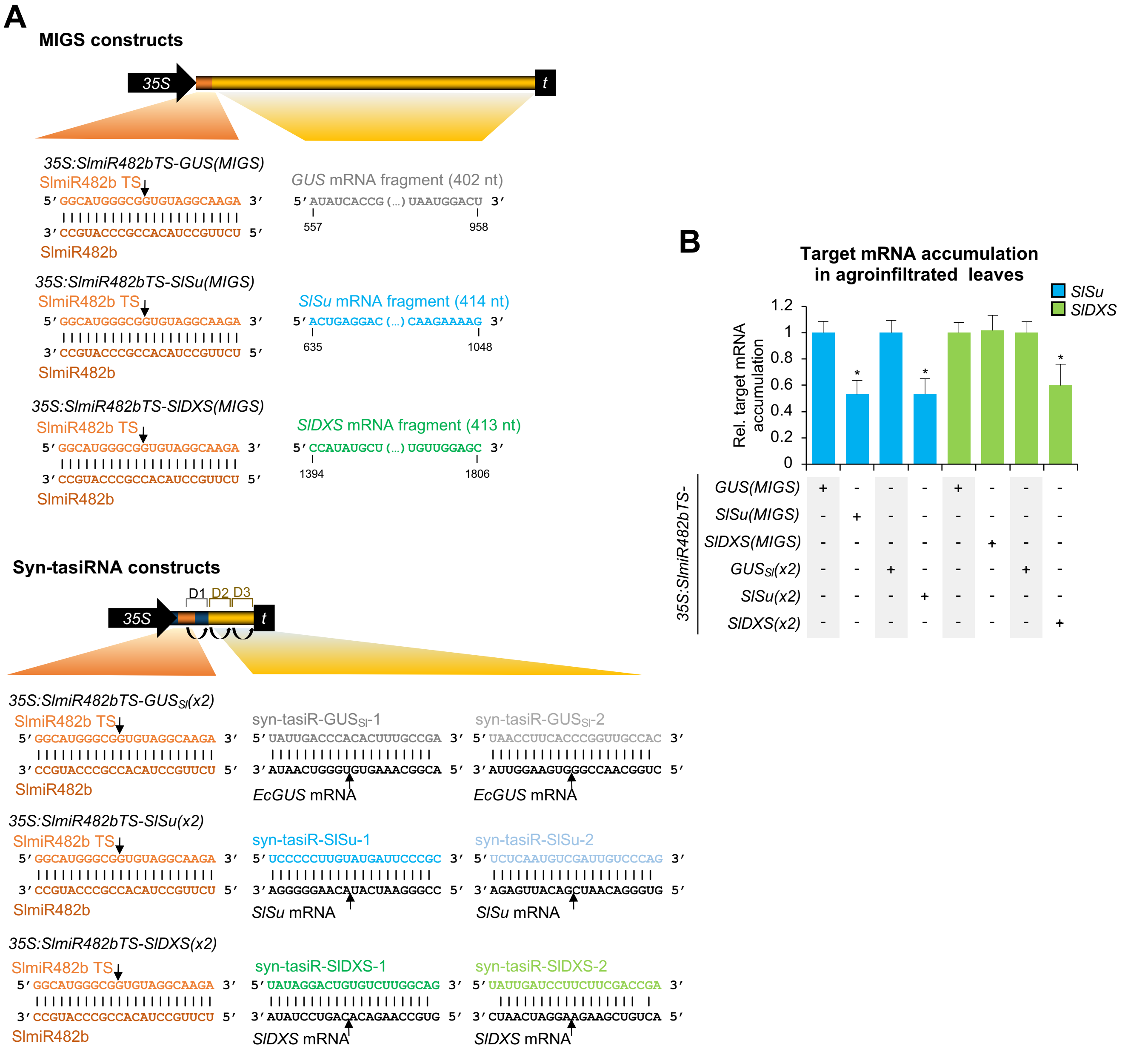

Figure S3. Comparative analysis of gene silencing induced by MIGS and syn-tasiRNAs in *Solanum lycopersicum*. (**A**) Organization of silencing constructs. Top: MIGS constructs. RNA fragments from *GUS*, *SlSu* and *SlDXS* mRNAs are in grey, blue and green, respectively. Fragment sizes and terminal nucleotide coordinates are indicated. Bottom: syn-tasiRNA constructs. Nucleotides corresponding to syn-tasiR-GUS$_{Sl}$(x2), syn-tasiR-SlSu and syn-tasiR-SlDXS are shown in grey, blue and green, respectively. Target mRNA nucleotides are in black. Sequences corresponding to SlmiR482b and its target site (TS) are shown in dark and light orange, respectively. Expected cleavage sites are indicated by arrows. (**B**)) Accumulation of *SlSu* and *SlDXS* mRNAs in tomato agroinfiltrated leaves. Data represent the mean (*n*=3) +SE relative expression of *SlSu* or *SlDXS* mRNA at two days post-agroinfiltration (dpa) after normalization to *ACTIN* (*SlACT*), as determined by RT–qPCR (*35S:SlmiR482bTS-GUS(MIGS)*=1 in all comparisons). Asterisks indicate significant differences from the control (P<0.05; pairwise Student's *t*-test comparison).

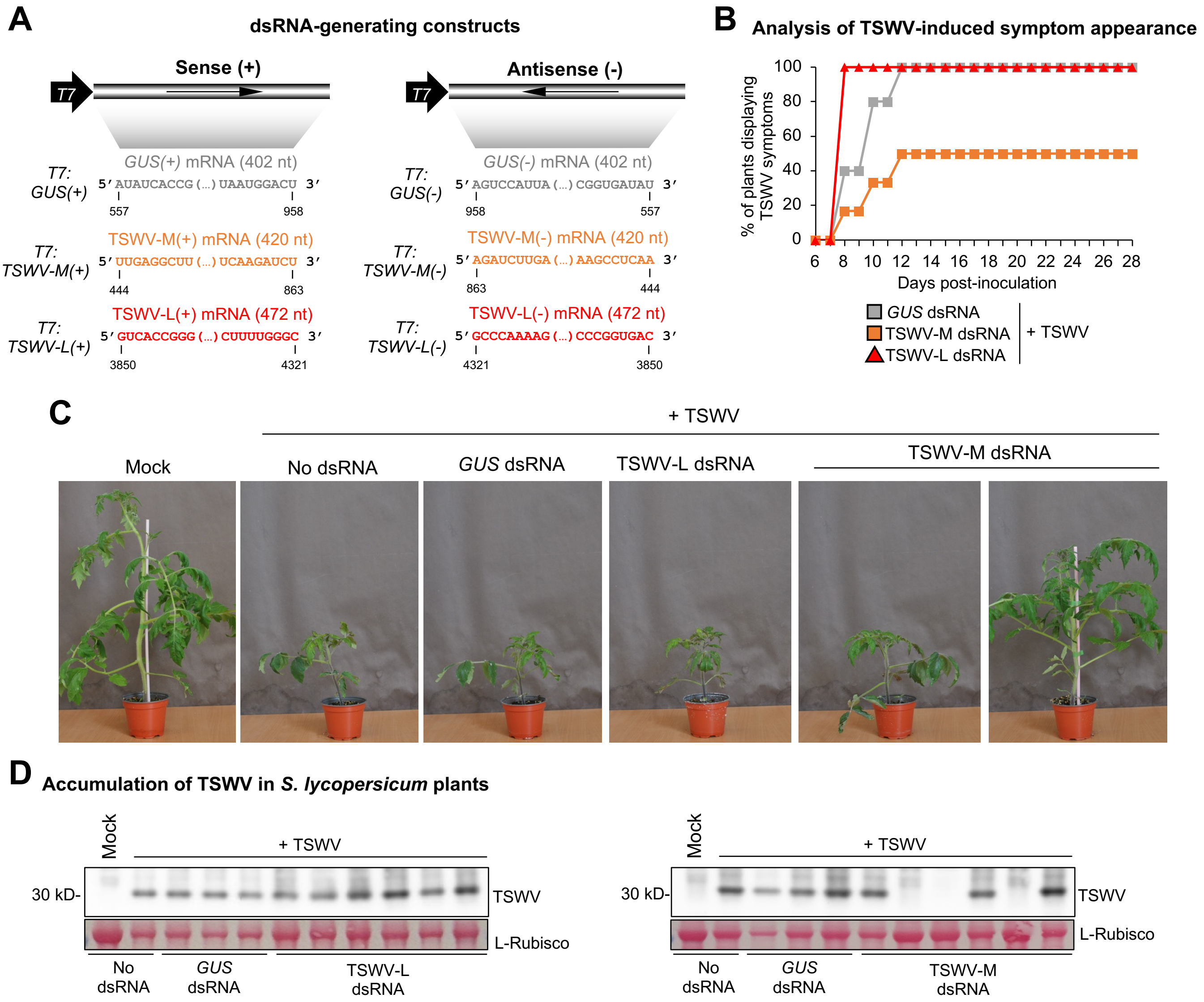

Figure S4. Functional analysis of dsRNA treatments against tomato spotted wilt virus (TSWV) in *S. lycopersicum*. (**A**) Schematic representation of T7-based constructs used for *in vitro* transcription. Nucleotides corresponding to *GUS*, TSWV-L and TSWV-M fragments are in grey, red and orange, respectively. (**B**) Two-dimensional line graph showing, for each six-plant set, the percentage of symptomatic plants recorded daily over 28 days. (**C**) Photographs taken at 20 days post-inoculation (dpi) of plants treated with the different dsRNAs and either inoculated with TSWV (+TSWV) or mock-inoculated. (**D**) Western blot detection of TSWV in protein extracts from apical leaves collected at 14 dpi. A Ponceau-stained membrane is provided as a loading control, highlighting the large subunit of Rubisco (ribulose1,5-biphosphate carboxylase/oxygenase).

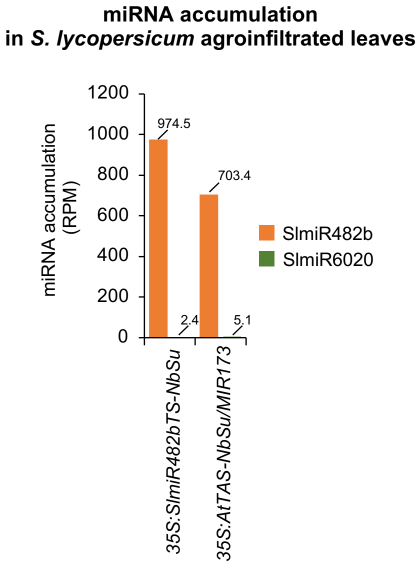

Figure S5. Analysis of SlmiR482b and SlmiR6020 presence in *Solanum lycopersicum* agroinfiltrated tissues. Left, bar graph showing the accumulation (reads per million, RPM) of SlmiR482b and SlmiR6020 revealed by high-throughput sequencing of small RNA libraries prepared from leaves agroinfiltrated with different constructs.

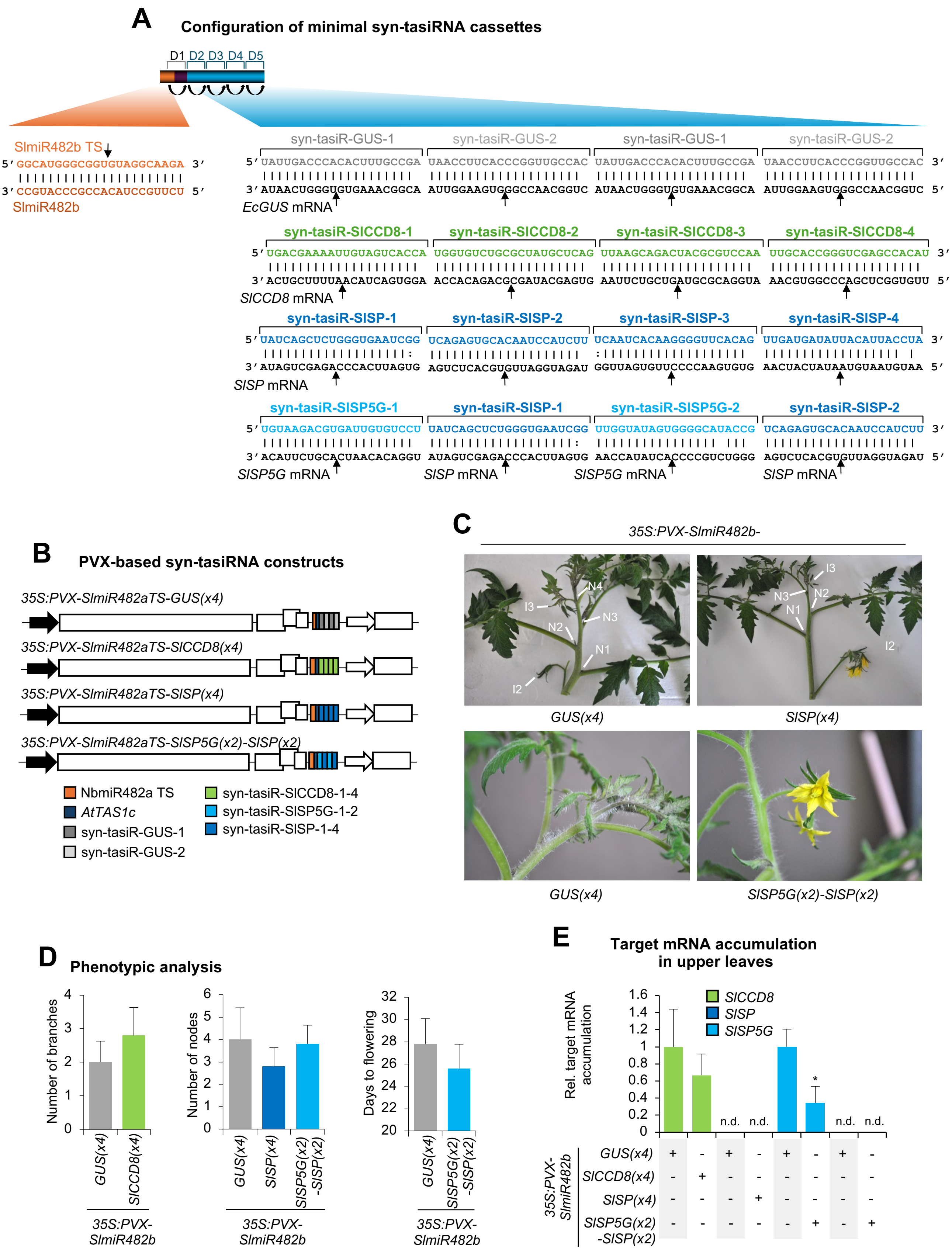

Figure S6. Functional analysis PVX-based syn-tasiR-VIGS silencing of *SlSP*/*SlSP5G* and *SlCCD8* silencing in *Solanum lycopersicum*. (**A**) Organization of minimal syn-tasiRNA precursors. Nucleotides (nt) corresponding to SlmiR482b target site (TS) and SlmiR482b and are shown in light and dark orange, respectively. Nucleotides corresponding to syn-tasiR-GUS, syn-tasiR-SlSP5G, syn-tasiR-SlSP and syn-tasiR-SlCCD8 are shown in grey, light blue, dark blue, and green, respectively. Arrows indicate the predicted cleavage sites for endogenous 22-nt miRNAs and syn-tasiRNAs. (**B**) Diagram of PVX-based constructs expressing anti-*SlCCD8,* anti-*SlSP,* anti-*SlSP5G* or anti-*GUS* syn-tasiRNAs. Color coding for the syn-tasiRNA sequences is consistent with panel (A). (**C**) Photographs of representative plants expressing syn-tasiR-SlSP or syn-tasiR-SlSP/syn-tasiR-SlSP5G through PVX-based syn-tasiR-VIGS. Top, nodes (N) and inflorescences (I) are indicated and numbered. Bottom, detail of the unopened and first opened inflorescences. (**D**) Phenotypic analysis of plants agroinoculated

Figure S6 (cont.) with syn-tasiR-VIGS constructs. Mean level (*n*=6) + SD of number or branches, number of nodes between the second and third inflorescence and days to flowering in plants agroinoculated with different contructs. Counting of branches and nodes was done at 40 days post-agroinoculation. (**E**) Accumulation of *SlSP5G* and *SlCCD8* mRNAs in tomato apical leaves. Data represent the mean (*n*=3) +SE relative expression of *SlCCD8* or *SlSP5G* mRNA at 14 days post-agroinoculation (dpa) after normalization to *ACTIN* (*SlACT*), as determined by RT–qPCR (*35S:SlmiR482bTS-GUS(x4)*=1 in all comparisons). Asterisk indicate significant differences from the control ($P<0.05$; pairwise Student's *t*-test comparison). n.d. refers to "not determined" due to insufficient expression.

Table S1. Name, sequence and use of oligonucleotides used in this study.

Name	Sequence	Type*	Construct/Aim
AC-50	CCGATTCACCCAGAGCTGATA	ssDNA	Probe to detect syn-tasiR-TSWV-2
AC-51	CGGTATGCCCCACTATACCAA	ssDNA	Probe to detect syn-tasiR-TSWV-3
AC-52	AAGATGGATTGTGCACTCTGA	ssDNA	Probe to detect syn-tasiR-TSWV-4
AC-55	AGGGGCCATGCTAATCTTCTC	ssDNA	Probe for U6 detection
AC-280	CTAGGCTGGGTTCGCAGGAGATGATGC	ssDNA	*SlACT* qPCR
AC-281	GTCTTTTTGACCCATACCCACCATCACAC	ssDNA	
AC-416	A+GGA+CAC+AAT+CAC+GTC+TTA+CA	ssLNA	Probe to detect syn-tasiR-TSWV-1
AC-417	G+CGG+GAA+GTC+CAC+CAC+GGT+TA	ssLNA	Probe for syn-tasiR-NbSu detection
AC-452	ATGGAGTTTTTGAGTCTTCTGC	ssDNA	*SlTIP41* qPCR
AC-453	GCTGCGTTTCTGGCTTAGG	ssDNA	
AC-525			
AC-654	GGGAATCAATCACAGTGTTGGC	ssDNA	syn-tasiRNA precursors detection
AC-655	GCTACTATGGCACGGGCTGTAC	ssDNA	
AC-657	ATGTCAGGCCTGTTCACTATCC	ssDNA	PVX detection
AC-658	TGGTGGTGGTAGAGTGACAAC	ssDNA	
AC-775	agaggtcagcaccagctagcGGCATGGGCGGTGTAGGCAAGATAGACCATTTATCCCCCTTGTATGATTCCCGCTCTCAATGTCGATTGTCCCAGagggtttgttaagtttccct	dsDNA	*35S:PVX-SlmiR482bTS-SlSu(x2)*
AC-776	agaggtcagcaccagctagcGGCATGGGCGGTGTAGGCAAGATAGACCATTTATATAGGACTGTGTCTTGGCAGTATTGATCCTTCTTCGACCGAagggtttgttaagtttccct	dsDNA	*35S:PVX-SlmiR482bTS-SlDXS(x2)*
AC-820	TGACCATGGATCTCCTGTTG	ssDNA	*SlDXS* qPCR
AC-821	GCCTCTCTGGTTTGTCCAAG	ssDNA	
AC-822	CTACACCCTTCACTTCTCGTTCC	ssDNA	*SlSu* qPCR
AC-823	AGTTTCTGAGCCTGTTCTTGAGC	ssDNA	
AC-824	TGTAGGCATGGGCGGTGTAGGCAAGATAGACCATTTATATTGACCCACACTTTGCCGA	ssDNA	*35S:SlmiR482bTS-GUS*$_{Sl}$
AC-825	AATGTCGGCAAAGTGTGGGTCAATATAAATGGTCTATCTTGCCTACACCGCCCATGCC	ssDNA	
AC-826	TGTAGGCATGGGCGGTGTAGGCAAGATAGACCATTTATGTATGACTCCCGGAATTCCA	ssDNA	*35S:SlmiR482bTS-NbSu*
AC-827	AATGTGGAATTCCGGGAGTCATACATAAATGGTCTATCTTGCCTACACCGCCCATGCC	ssDNA	
AC-828	TGTAAAAGATACTCGGAAAACATTTATAGACCATTTATATTGACCCACACTTTGCCGA	ssDNA	*35S:SlmiR6020TS-GUS*$_{Sl}$
AC-829	AATGTCGGCAAAGTGTGGGTCAATATAAATGGTCTATAAATGTTTTCCGAGTATCTTT	ssDNA	
AC-830	TGTAAAAGATACTCGGAAAACATTTATAGACCATTTATGTATGACTCCCGGAATTCCA	ssDNA	*35S:SlmiR6020TS-NbSu*
AC-831	AATGTGGAATTCCGGGAGTCATACATAAATGGTCTATAAATGTTTTCCGAGTATCTTT	ssDNA	
AC-832	GTTGGTCGTGTGGTAGGGGA	ssDNA	*SlSFT* qPCR
AC-833	GCCTAAGCTCGCATCCATTA	ssDNA	
AC-874	G+CGG+GAA+TCA+TAC+AAG+GGG+GA	ssLNA	Probe to detect syn-tasiR-SlSu-1
AC-875	C+TGG+GAC+AAT+CGA+CAT+TGA+GA	ssLNA	Probe to detect syn-tasiR-SlSu-2
AC-876	C+TGC+CAA+GAC+ACA+GTC+CTA+TA	ssLNA	Probe to detect syn-tasiR-SlDXS-1
AC-877	T+CGG+TCG+AAG+AAG+GAT+CAA+TA	ssLNA	Probe to detect syn-tasiR-SlDXS-2
AC-892	TGTAGGCATGGGCGGTGTAGGCAAGATAGACCATTTATCCCCCTTGTATGATTCCCGCTCTCAATGTCGATTGTCCCAG	ssDNA	*35S:SlmiR482bTS-SlSu(x2)*
AC-893	AATGCTGGGACAATCGACATTGAGAGCGGGAATCATACAAGGGGGATAAATGGTCTATCTTGCCTACACCGCCCATGCC	ssDNA	
AC-894	TGTAGGCATGGGCGGTGTAGGCAAGATAGACCATTTATATAGGACTGTGTCTTGGCAGTATTGATCCTTCTTCGACCGA	ssDNA	*35S:SlmiR482bTS-SlDXS(x2)*
AC-895	AATGTCGGTCGAAGAAGGATCAATACTGCCAAGACACAGTCCTATATAAATGGTCTATCTTGCCTACACCGCCCATGCC	ssDNA	

AC-902	TGTAGGCATGGGCGGTGTAGGCAAGATAGA CCATTTAaGAGACCggtctcATCGG	ssDNA	*pENTR-SlmiR482bTS-BB*, *pMDC32B-SlmiR482bTS-BB*
AC-903	AATGCCGATgagaccGGTCTCtTAAATGGTCTA TCTTGCCTACACCGCCCATGCC	ssDNA	
AC-909	TTTATGCATACACTGTTTGCCGGCT	ssDNA	*35S:SlmiR482bTS-SlSFT*
AC-910	CCGAAGCCGGCAAACAGTGTATGCA	ssDNA	
AC-915	TGTAGGCATGGGCGGTGTAGGCAAGATAGA CCATTTATATTGACCCACACTTTGCCGATAA CCTTCACCCGGTTGCCAC	ssDNA	*35S:SlmiR482bTS-GUS*$_{Sl}$*(x2)*
AC-916	AATGGTGGCAACCGGGTGAAGGTTATCGGC AAAGTGTGGGTCAATATAAATGGTCTATCT TGCCTACACCGCCCATGCC	ssDNA	
AC-993	agaggtcagcaccagctagcGGCATGGGCGGTGTAGG CAAGATAGACCATTTATATTGACCCACACT TTGCCGATAACCTTCACCCGGTTGCCACTAT TGACCCACACTTTGCCGATAACCTTCACCC GGTTGCCACagggtttgttaagtttccct	dsDNA	*35S:PVX-SlmiR482bTS-GUS*$_{Sl}$*(x4)*
AC-1170	GTCACCGGGTCTGTAGATTTCCTAG	ssDNA	*T7:TSWV-L(+)*, *T7:TSWV-L(-)*
AC-1171	GCCCAAAAGTTCCTTGAGCCTTTC	ssDNA	
AC-1205	agaggtcagcaccagctagcGTGATTTTTCTCTACAAG CGAATAGACCATTTATGTAAGACGTGATTG TGTCCTTATCAGCTCTGGGTGAATCGGTTGG TATAGTGGGGCATACCGTCAGAGTGCACAA TCCATCTTagggtttgttaagtttccct	dsDNA	*35S:PVX-AtmiR173aTS-TSWV(x4)*
AC-1224	A+GCC+GGC+AAA+CAG+TGT+ATG+CA	LNA	Probe to detect syn-tasiR-SlSFT
AC-1250	AGGGTTGAAGTTGGAGGAGATGACC	ssDNA	*SlSFT* control PCR (5'RLM-RACE)
AC-1251	GTAAGAGAAGTAGTAGATATTGGTGGTT	ssDNA	*SlSFT* round 1 PCR (5'RLM-RACE)
AC-1252	CGTCCACCACTGCCACTCTCTCTTTG	ssDNA	*SlSFT* control PCR (5'RLM-RACE)<br>*SlSFT* round 2 PCR (5'RLM-RACE)
AC-1325	agaggtcagcaccagctagcGGCATGGGCGGTGTAGG CAAGATAGACCATTTATTTCATACCCAACC ACTTCTGTTTGTCGATATATCAACGCCATCA ATCACAAGGGGTTCACAGTTGATGATATTA CATTACCTAagggtttgttaagtttccct	dsDNA	*35S:PVX-SlmiR482bTS-SlSP(x4)*
AC-1327	agaggtcagcaccagctagcGGCATGGGCGGTGTAGG CAAGATAGACCATTTATGTCCGGTGCATCG ATGGCAATTTCATACCCAACCACTTCTGTCG AGAACGGATCGCACCCCCTTTGTCGATATA TCAACGCCAagggtttgttaagtttccct	dsDNA	*35S:PVX-SlmiR482bTS-SlSP5G(x2)-SlSP(x2)*
AC-1328	agaggtcagcaccagctagcGGCATGGGCGGTGTAGG CAAGATAGACCATTTATGACGAAAATTGTA GTCACCATGGTGTCTGCGCTATGCTCAGTTA AGCAGACTACGCGTCCAATTGCACCGGGTC GAGCCACATagggtttgttaagtttccct	dsDNA	*35S:PVX-SlmiR482bTS-SlCCD8(x4)*
AC-1329	caccGGCATGGGCGGTGTAGGCAAGAATATC ACCGTGGTGACGCATG	ssDNA	*35S:SlmiR482bTS-GUS*$_{Sl}$*(MIGS)*
AC-1330	AGTCCATTAATGCGTGGTCGTG	ssDNA	*35S:SlmiR482bTS-GUS*$_{Sl}$*(MIGS)*, *T7:GUS(+)*, *T7:GUS(-)*
AC-1331	caccGGCATGGGCGGTGTAGGCAAGAACTGA GGACAGGGTGTGTGGG	ssDNA	*35S:SlmiR482bTS-SlSu(MIGS)*
AC-1332	CTTTTCTTGCTCCCCCTTGTATG	ssDNA	
AC-1333	caccGGCATGGGCGGTGTAGGCAAGACCATA TGCTGAGAGAGCTGCAG	ssDNA	*35S:SlmiR482bTS-SlDXS(MIGS)*
AC-1334	GCTCCAACAAGACCTGCTCTG	ssDNA	
AC-1335	ATATCACCGTGGTGACGCATG	ssDNA	*T7:GUS(+)*, *T7:GUS(-)*
AC-1338	TCAAGATCTGTCCAACCTGGG	ssDNA	*T7:TSWV-M(+)*, *T7:TSWV-M(-)*
AC-1339	AAGCCTCAATGAGTGCTTGAG	ssDNA	
GeneRacer 5' Oligo	CGACTGGAGCACGAGGACACTGA	ssDNA	*SlSFT* round 1 PCR (5'RLM-RACE)
GeneRacer 5' Nested Oligo	GGACACTGACATGGACTGAAGGAGTA	ssDNA	*SlSFT* round 2 PCR (5'RLM-RACE)

GeneRacer Oligo dT	GCTGTCAACGATACGCTACGTAACGGCATG ACAGTGTTTTTTTTTTTTTTTTTTTTTTTT	ssDNA	cDNA synthesis (5'RLM-RACE)
GeneRacer RNA Oligo Adapter	CGACUGGAGCACGAGGACACUGACAUGGA CUGAAGGAGUAGAAA	ssRNA	RNA ligation (5'RLM-RACE)

*ssDNA: single-stranded DNA; dsDNA: double-stranded DNA; LNA: locked nucleic acid;ssRNA, single-stranded RNA.

Text S1. Protocol to design and clone syn-tasiRNAs downstream the 3'D1[+] position in *Bsa*I/*ccd*B-based ('B/c') vectors *pENTR-SlmiR482bTS-B/c* and *pMDC32B-SlmiR482bTS-B/c*.

1. Selection of the syn-tasiRNA sequence(s)

Use the Syn-tasiRNA Designer app from the P-SAMS webtool at http://p-sams.carringtonlab.org/syntasi/designer.

2. Design of syn-tasiRNA oligonucleotides for cloning

Next are described some designs for cloning two syn-tasiRNAs in tandem downstream the 3'D1[+] position.

Use vectors *pENTR-SlmiR482bTS-B/c* or *pMDC32-SlmiR482bTS-B/c* and order the following oligos:

-Forward oligonucleotide (46 b):

TTTA$X_1X_2X_3X_4X_5X_6X_7X_8X_9X_{10}X_{11}X_{12}X_{13}X_{14}X_{15}X_{16}X_{17}X_{18}X_{19}X_{20}X_{21}X_1X_2X_3X_4X_5X_6X_7X_8X_9X_{10}X_{11}X_{12}$
$X_{13}X_{14}X_{15}X_{16}X_{17}X_{18}X_{19}X_{20}X_{21}$

-Reverse oligonucleotide (46 b):

CCGA$Y_{21}Y_{20}Y_{19}Y_{18}Y_{17}Y_{16}Y_{15}Y_{14}Y_{13}Y_{12}Y_{11}Y_{10}Y_9Y_8Y_7Y_6Y_5Y_4Y_3Y_2Y_1Y_{21}Y_{20}Y_{19}Y_{18}Y_{17}$
$Y_{16}Y_{15}Y_{14}Y_{13}Y_{12}Y_{11}Y_{10}Y_9Y_8Y_7Y_6Y_5Y_4Y_3Y_2Y_1$

Where:

$X_1X_2X_3X_4X_5X_6X_7X_8X_9X_{10}X_{11}X_{12}X_{13}X_{14}X_{15}X_{16}X_{17}X_{18}X_{19}X_{20}X_{21}$=syn-tasiRNA-1 sequence

$X_1X_2X_3X_4X_5X_6X_7X_8X_9X_{10}X_{11}X_{12}X_{13}X_{14}X_{15}X_{16}X_{17}X_{18}X_{19}X_{20}X_{21}$=syn-tasiRNA-2 sequence

$Y_{21}Y_{20}Y_{19}Y_{18}Y_{17}Y_{16}Y_{15}Y_{14}Y_{13}Y_{12}Y_{11}Y_{10}Y_9Y_8Y_7Y_6Y_5Y_4Y_3Y_2Y_1$=syn-tasiRNA-1 reverse-complement sequence

$Y_{21}Y_{20}Y_{19}Y_{18}Y_{17}Y_{16}Y_{15}Y_{14}Y_{13}Y_{12}Y_{11}Y_{10}Y_9Y_8Y_7Y_6Y_5Y_4Y_3Y_2Y_1$=syn-tasiRNA-2 reverse-complement sequence

Example

The sequences of the two oligonucleotides to clone syn-tasiRNAs 'syn-tasiR-TRY' (TCCCATTCGATACTGCTCGCC) and 'syn-tasiR-Ft' (TTGGTTATAAAGGAAGAGGCC) in positions 3'D2[+] and 3'D3[+], respectively, of minimal precursors included in *SlmiR482bTS*-based B/c vectors are:

-Forward oligonucleotide (46 b):

TTTATCCCATTCGATACTGCTCGCCTTGGTTATAAAGGAAGAGGCC

-Reverse oligonucleotide (46 b):

CCGAGGCCTCTTCCTTTATAACCAAGGCGAGCAGTATCGAATGGGA

3. Cloning of the syn-tasiRNA sequence(s) in B/c-based vectors

Notes:

-New available -B/c vectors are listed in Table I at the end of the section.

-B/c-*based vectors must be propagated in a* ccd*B resistant* E. coli *strain such as DB3.1.*

-Alternatively, Bsa*I digestion of the B/c vector and subsequent ligation of the amiRNA oligonucleotide insert can be done in separate reactions*

3.1. Oligonucleotide annealing

-Dilute sense oligonucleotide and antisense oligonucleotide in sterile H_2O to a final concentration of 100 µM.

-Prepare Oligo Annealing Buffer:

60 mM Tris-HCl (pH 7.5)

500 mM NaCl

60 mM $MgCl_2$

10 mM DTT

Note: *Prepare 1 ml aliquots of Oligo Annealing Buffer and store at -20^{0}C.*

-Assemble the annealing reaction in a PCR tube as described below:

Forward oligonucleotide (100 µM)	2 µL
Reverse oligonucleotide (100 µM)	2 µL
Oligo Annealing Buffer	46 µL
Total volume	50 µL

The final concentration of each oligonucleotide is 4 µM.

-Use a thermocycler to heat the annealing reaction 5 min at 94°C and then cool down (0.05ºC/sec) to 20°C.

-Dilute the annealed oligonucleotides just prior to assembling the digestion-ligation reaction as described below:

Annealed oligonucleotides	3 µL
dH_2O	37 µL
Total volume	40 µL

The final concentration of each oligonucleotide is 0.15 µM.

***Note**: Do not store the diluted oligonucleotides.*

3.2. Digestion-ligation reaction

- Assemble the digestion-ligation reaction as described below:

B/c vector (x ug/uL)	Y µL (50 ng)
Diluted annealed oligonucleotides	1 µL
10x T4 DNA ligase buffer	1 µL
T4 DNA ligase (400 U/µL)	1 µL
*Bsa*I (10U/ µL, NEB)	1 µL
dH_2O	to 10 µL
Total volume	10 µL

Prepare a negative control reaction lacking *Bsa*I.

-Mix the reactions by pipetting. Incubate the reactions at room temperature for 5 minutes at 37°C.

3.3. *E.coli* transformation and analysis of transformants

-Transform 1-5 ul of the digestion-ligation reaction into an *E. coli* strain that doesn't have *ccd*B resistance (e.g. DH10B, TOP10, …) to do counter-selection.

-Pick two colonies/construct, grow LB-Kan (100 mg/ml) cultures and purify plasmids.

-Sequence with appropriate primers: M13-F (CCCAGTCACGACGTTGTAAAACGACGG) and M13-R (CAGAGCTGCCAGGAAACAGCTATGACC) for *pENTR*-based vectors; attB1 (ACAAGTTTGTACAAAAAAGCAGGCT) and attB2 (ACCACTTTGTACAAGAAAGCTGGGT) primers for *pMDC32B*-based vectors).

Table I: *Bsa*I/*ccd*B-based ('B/c') vectors for direct cloning of syn-tasiRNAs downstream position 3'D1[+] in minimal precursor including SlmiR482b TS.

Vector	Small RNA expressed	Bacterial antibiotic resistance	Plant antibiotic resistance	GATEWAY use	Backbone	Promoter of syn-tasiRNA cassette	Terminator of syn-tasiRNA cassette	Plant species tested
pENTR-SlmiR482bTS-B/c	syn-tasiRNAs	Kanamycin	–	Donor	*pENTR*	–	–	–
pMDC32B-SlmiR482bTS-B/c	syn-tasiRNAs	Kanamycin Hygromycin	Hygromycin	–	*pMDC32*	*CaMV* 2x35S	*Nos*	*S. lycopersicum*

Text S2. Protocol to generate PVX-based syn-tasiRNA constructs.

1. Preparation of the dsDNA syn-tasiRNA insert

Design and order a dsDNA (eg. ultramer duplex in IDT) including the sequences of your syn-tasiRNA(s) (2 in the following example) following the 22-nt miRNA target site of interest, as follows:

agaggtcagcaccagctagc$X_1X_2X_3X_4X_5X_6X_7X_8X_9X_{10}X_{11}X_{12}X_{13}X_{14}X_{15}X_{16}X_{17}X_{18}X_{19}X_{20}X_{21}X_{22}$TAGAC CATTTA$X_1X_2X_3X_4X_5X_6X_7X_8X_9X_{10}X_{11}X_{12}X_{13}X_{14}X_{15}X_{16}X_{17}X_{18}X_{19}X_{20}X_{21}X_1X_2X_3X_4X_5X_6X_7X_8X_9X_{10}X_{11}X_{12}X_{13}X_{14}X_{15}X_{16}X_{17}X_{18}X_{19}X_{20}X_{21}$agggtttgttaagtttccct

Where:

- -X is a DNA base of the 22-nt miRNA target site sequence, and the subscript number is the base position
- -X is a DNA base of the syn-tasiRNA-1 sequence, and the subscript number is the base position in the syn-tasiRNA* 21-mer
- -X is a DNA base of the syn-tasiRNA-2 sequence, and the subscript number is the base position in the syn-tasiRNA 21-mer
- -x is a DNA base of the PVX sequence, required for Gibson-based assembly
- -X is a DNA base of the *AtTAS1c* sequence

Note that:

- -In general, **X_1=T** and **X_1=T** for amiRNA association with AGO1.

Fragment #1 (syn-tasiRNA precursor) is ready.

2. Preparation of the vector

-Digest *pLB-PVX* with *Mlu*I.

-Gel purify the 9921 bp band corresonding to linearized plasmid.

-Quantify 1 ul in Nanodrop.

Fragment #2 (backbone vector) is ready.

3. Assembly

-Assemble the Gibbson reaction as described below:

Fragment 1 (dsDNA insert)[a]	
Fragment 2 (vector)[b,c,d]	
GeneArt Gibson Assembly HiFI Master Mix	5 µL
dH_2O	to 10 µL

Total volume 10 µL

[a]The optimal amount of vector is between 50-100 ng

[b]Insert/vector molar excess is between 2-3.

[c]Total DNA amount is between 0.02-0.5 pmol

[d]Mass to moles conversions can be calculated here:

http://nebiocalculator.neb.com/#!/ssdnaamt

-Incubate reactions at 50ºC for 1h.

-Clean up reactions with a column (e.g. Zymo Research)

-Transform 1-4 µL in *E. coli* DH5α

-Plate in L-Kan plates and incubate 16h at 37ºC

4. Clone verification

-Pick several colonies and grow in liquid LB-Kan 16h at 37ºC, and purify plasmids.

-Digest candidate clones with *Apa*I+*Xho*I

Good clones: 8595 + **1409** bp

Bad clones (empty *pLB-PVX*): 9921 bp + **1738** bp

-Confirm insert sequence by Sanger sequencing with forward and reverse oligos AC-654 (`GGGAATCAATCACAGTGTTGGC`) and/or AC-655 (`GCTACTATGGCACGGGCTGTAC`), respectively.

Text S3. DNA sequence in FASTA format of all precursors used to express syn-tasiRNAs in plants.

1. *AtTAS1c*-based precursors

>AtTAS1c-NbSu
AAACCTAAACCTAAACGGCTAAGCCCGACGTCAAATACCAAAAAGAGAAAAACAAGAGCGCCGTCAAGCTCTGCAAATACGATCTGTAAG
TCCATCTTAACACAAAAGTGAGATGGGTTCTTAGATCATGTTCCGCCGTTAGATCGAGTCATGGTCTTGTCTCATAGAAAGGTACTTTCG
TTTACTTCTTTTGAGTATCGAGTAGAGCGTCGTCTATAGTTAGTTTGAGATTGCGTTTGTCAGAAGTTAGGTTCAATGTCCCGGTCCAAT
TTTCACCAGCCATGTGTCAGTTTCGTTCCTTCCCGTCCTCTTCTTTGATTTCGTTGGGTTACGGATGTTTTCGAGATGAAACAGCATTGT
TTTGTTGTGATTTTTCTCTACAAGCGAATAGACCATTTATGTATGACTCCCGGAATTCCATCGGTGGATCTTAGAAAATTATTCTAAGTC
CAACATAGCGTATTCTAAGTTCAACATATCGACGAACTAGAAAAGACATTGGACATATTCCAGGATATGCAAAAGAAAACAATGAATATT
GTTTTGAATGTGTTCAAGTAAATGAGATTTTCAAGTCGTCTAAAGAACAGTTGCTAATACAGTTACTTATTTCAATAAATAATTGGTTCT
AATAATACAAAACATATTCGAGGATATGCAGAAAAAAAGATGTTTGTTATTTTGAAAAGCTTGAGTAGTTTCTCTCCGAGGTGTAGCGAA
GAAGCATCATCTACTTTGTAATGTAATTTTCTTTATGTTTTCACTTTGTAATTTTATTTGTGTTAATGTACCATGGCCGATATCGGTTTT
ATTGAAAGAAAATTTATGTTACTTCTGTTTTGGCTTTGCAATCAGTTATGCTAGTTTTCTTATACCCTTTCGTAAGCTTCCTAAGGAATC
GTTCATTGATTTCCACTGCTTCATTGTATATTAAAACTTTACAACTGTATCGACCATCATATAATTCTGGGTCAAGAGATGAAAATAGAA
CACCACATCGTAAAGTGAAAT

AtTAS1c
AtmiR173a TS
syn-tasiR-NbSu

2. Minimal syn-tasiRNA precursors

>SlmiR482bTS-GUS$_{Nb}$
GGCATGGGCGGTGTAGGCAAGATAGACCATTTATATTGACCCACACTTTGCCGA

AtTAS1c
SlmiR482b TS
syn-tasiR-GUS$_{Nb}$

>SlmiR482bTS-NbSu
GGCATGGGCGGTGTAGGCAAGATAGACCATTTATGTATGACTCCCGGAATTCCA

AtTAS1c
SlmiR482b TS
syn-tasiR-NbSu

>SlmiR6020TS-GUS$_{Nb}$
AAAGATACTCGGAAAACATTTATAGACCATTTATATTGACCCACACTTTGCCGA

AtTAS1c
SlmiR6020 TS
syn-tasiR-GUS$_{Nb}$

>SlmiR6020TS-NbSu
AAAGATACTCGGAAAACATTTATAGACCATTTATGTATGACTCCCGGAATTCCA

AtTAS1c
SlmiR6020 TS
syn-tasiR-NbSu

>SlmiR482bTS-SlSFT
GGCATGGGCGGTGTAGGCAAGATAGACCATTTATGCATACACTGTTTGCCGGCT

AtTAS1c
SlmiR482b TS
syn-tasiR-SlSFT

>SlmiR482bTS-GUS$_{S1}$(x4)

GGCATGGGCGGTGTAGGCAAGATAGACCATTTATATTGACCCACACTTTGCCGATAACCTTCACCCGGTTGCCACTATTGACCC
ACACTTTGCCGATAACCTTCACCCGGTTGCCAC

AtTAS1c
SlmiR482b TS
syn-tasiR-GUS$_{S1}$
syn-tasiR-GUS$_{S1}$-2

>SlmiR482bTS-TSWV(x4)

GGCATGGGCGGTGTAGGCAAGATAGACCATTTATGTAAGACGTGATTGTGTCCTTATCAGCTCTGGGTGAATCGGTTGGTATAG
TGGGGCATACCGTCAGAGTGCACAATCCATCTT

AtTAS1c
SlmiR482b TS
syn-tasiR-TSWV-1
syn-tasiR-TSWV-2
syn-tasiR-TSWV-3
syn-tasiR-TSWV-4

>AtmiR173a-TSWV(x4)

GTGATTTTTCTCTACAAGCGAATAGACCATTTATGTAAGACGTGATTGTGTCCTTATCAGCTCTGGGTGAATCGGTTGGTATAG
TGGGGCATACCGTCAGAGTGCACAATCCATCTT

AtTAS1c
AtmiR173a TS
syn-tasiR-TSWV-1
syn-tasiR-TSWV-2
syn-tasiR-TSWV-3
syn-tasiR-TSWV-4

>SlmiR482bTS-SlGUS$_{S1}$(x2)

GGCATGGGCGGTGTAGGCAAGATAGACCATTTATATTGACCCACACTTTGCCGATAACCTTCACCCGGTTGCCAC

AtTAS1c
SlmiR482b TS
syn-tasiR-GUS$_{S1}$-1
syn-tasiR-GUS$_{S1}$-2

>SlmiR482bTS-SlSu(x2)

GGCATGGGCGGTGTAGGCAAGATAGACCATTTATCCCCCTTGTATGATTCCCGCTCTCAATGTCGATTGTCCCAG

AtTAS1c
SlmiR482b TS
syn-tasiR-SlSu-1
syn-tasiR-SlSu-2

>SlmiR482bTS-SlDXS(x2)

GGCATGGGCGGTGTAGGCAAGATAGACCATTTATATAGGACTGTGTCTTGGCAGTATTGATCCTTCTTCGACCGA

AtTAS1c
SlmiR482b TS
syn-tasiR-SlDXS1-1
syn-tasiR-SlDXS1-2

Text S4. DNA sequence of *Bsa*I-*ccd*B-based (B/c) vectors used for direct cloning of syn-tasiRNAs.

>pENTR-SlmiR482bTS-B/c (4082 bp)

CTTTCCTGCGTTATCCCCTGATTCTGTGGATAACCGTATTACCGCCTTTGAGTGAGCTGATACCGCTCGCCGCAGCCGAACGACCGAGCG
CAGCGAGTCAGTGAGCGAGGAAGCGGAAGAGCGCCCAATACGCAAACCGCCTCTCCCCGCGCGTTGGCCGATTCATTAATGCAGCTGGCA
CGACAGGTTTCCCGACTGGAAAGCGGGCAGTGAGCGCAACGCAATTAATACGCGTACCGCTAGCCAGGAAGAGTTTGTAGAAACGCAAAA
AGGCCATCCGTCAGGATGGCCTTCTGCTTAGTTTGATGCCTGGCAGTTTATGGCGGGCGTCCTGCCCGCCACCCTCCGGGCCGTTGCTTC
ACAACGTTCAAATCCGCTCCCGGCGGATTTGTCCTACTCAGGAGAGCGTTCACCGACAAACAACAGATAAAACGAAAGGCCCAGTCTTCC
GACTGAGCCTTTCGTTTTATTTGATGCCTGGCAGTTCCCTACTCTCGCGTTAACGCTAGCATGGATGTTTTCCCAGTCACGACGTTGTAA
AACGACGGCCAGTCTTAAGCTCGGGCCCCAAATAATGATTTTATTTTGACTGATAGTGACCTGTTCGTTGCAACAAATTGATGAGCAATG
CTTTTTTATAATGCCAACTTTGTACAAAAAAGCAGGCTCCGCGGCCGCCCCCTTCACCTGTA**GGCATGGGCGGTGTAGGCAAGATAGACC**
ATTTAAGAGACCATTAGGCACCCCAGGCTTTACACTTTATGCTTCCGGCTCGTATAATGTGTGGATTTTGAGTTAGGAGCCGTCGAGATT
TTCAGGAGCTAAGGAAGCTAAAATGGAGAAAAAAATCACTGGATATACCACCGTTGATATATCCCAATGGCATCGTAAAGAACATTTTGA
GGCATTTCAGTCAGTTGCTCAATGTACCTATAACCAGACCGTTCAGCTGGATATTACGGCCTTTTTAAAGACCGTAAAGAAAAATAAGCA
CAAGTTTTATCCGGCCTTTATTCACATTCTTGCCCGCCTGATGAATGCTCATCCGGAGTTCCGTATGGCAATGAAAGACGGTGAGCTGGT
GATATGGGATAGTGTTCACCCTTGTTACACCGTTTTCCATGAGCAAACTGAAACGTTTTCATCGCTCTGGAGTGAATACCACGACGATTT
CCGGCAGTTTCTACACATATATTCGCAAGATGTGGCGTGTTACGGTGAAAACCTGGCCTATTTCCCTAAAGGGTTTATTGAGAATATGTT
TTTCGTCTCAGCCAATCCCTGGGTGAGTTTCACCAGTTTTGATTTAAACGTGGCCAATATGGACAACTTCTTCGCCCCCGTTTTCACCAT
GGGCAAATATTATACGCAAGGCGACAAGGTGCTGATGCCGCTGGCGATTCAGGTTCATCATGCCGTTTGTGATGGCTTCCATGTCGGCAG
AATGCTTAATGAATTACAACAGTACTGCGATGAGTGGCAGGGCGGGGCGTAAACGCGTGGAGCCGGCTTACTAAAAGCCAGATAACAGTA
TGCGTATTTGCGCGCTGATTTTTGCGGTATAAGAATATATACTGATATGTATACCCGAAGTATGTCAAAAAGAGGTATGCTATGAAGCAG
CGTATTACAGTGACAGTTGACAGCGACAGCTATCAGTTGCTCAAGGCATATATGATGTCAATATCTCCGGTCTGGTAAGCACAACCATGC
AGAATGAAGCCCGTCGTCTGCGTGCCGAACGCTGGAAAGCGGAAAATCAGGAAGGGATGGCTGAGGTCGCCCGGTTTATTGAAATGAACG
GCTCTTTTGCTGACGAGAACAGGGGCTGGTGAAATGCAGTTTAAGGTTTACACCTATAAAAGAGAGAGCCGTTATCGTCTGTTTGTGGAT
GTACAGAGTGATATTATTGACACGCCCGGCCGACGGATGGTGATCCCCCTGGCCAGTGCACGTCTGCTGTCAGATAAAGTCTCCCGTGAA
CTTTACCCGGTGGTGCATATCGGGGATGAAAGCTGGCGCATGATGACCACCGATATGGCCAGTGTGCCGGTTTCCGTTATCGGGGAAGAA
GTGGCTGATCTCAGCCACCGCGAAAATGACATCAAAAACGCCATTAACCTGATGTTCTGGGGAATATAAATGTCAGGCTCCCTTATACAC
AGCCAGTCTGCACCTCGACGGTCTCACATTAAGGGTGGGCGCGCCGACCCAGCTTTCTTGTACAAAGTTGGCATTATAAGAAAGCATTGC
TTATCAATTTGTTGCAACGAACAGGTCACTATCAGTCAAAATAAAATCATTATTTGCCATCCAGCTGATATCCCCTATAGTGAGTCGTAT
TACATGGTCATAGCTGTTTCCTGGCAGCTCTGGCCCGTGTCTCAAAATCTCTGATGTTACATTGCACAAGATAAAAATATATCATCATGA
ACAATAAAACTGTCTGCTTACATAAACAGTAATACAAGGGGTGTTATGAGCCATATTCAACGGGAAACGTCGAGGCCGCGATTAAATTCC
AACATGGATGCTGATTTATATGGGTATAAATGGGCTCGCGATAATGTCGGGCAATCAGGTGCGACAATCTATCGCTTGTATGGGAAGCCC
GATGCGCCAGAGTTGTTTCTGAAACATGGCAAAGGTAGCGTTGCCAATGATGTTACAGATGAGATGGTCAGACTAAACTGGCTGACGGAA
TTTATGCCTCTTCCGACCATCAAGCATTTTATCCGTACTCCTGATGATGCATGGTTACTCACCACTGCGATCCCCGGAAAAACAGCATTC
CAGGTATTAGAAGAATATCCTGATTCAGGTGAAAATATTGTTGATGCGCTGGCAGTGTTCCTGCGCCGGTTGCATTCGATTCCTGTTTGT
AATTGTCCTTTTAACAGCGATCGCGTATTTCGTCTCGCTCAGGCGCAATCACGAATGAATAACGGTTTGGTTGATGCGAGTGATTTTGAT
GACGAGCGTAATGGCTGGCCTGTTGAACAAGTCTGGAAAGAAATGCATAAACTTTTGCCATTCTCACCGGATTCAGTCGTCACTCATGGT
GATTTCTCACTTGATAACCTTATTTTTGACGAGGGGAAATTAATAGGTTGTATTGATGTTGGACGAGTCGGAATCGCAGACCGATACCAG
GATCTTGCCATCCTATGGAACTGCCTCGGTGAGTTTTCTCCTTCATTACAGAAACGGCTTTTTCAAAAATATGGTATTGATAATCCTGAT
ATGAATAAATTGCAGTTTCATTTGATGCTCGATGAGTTTTTCTAATCAGAATTGGTTAATTGGTTGTAACACTGGCAGAGCATTACGCTG
ACTTGACGGGACGGCGCAAGCTCATGACCAAAATCCCTTAACGTGAGTTACGCGTCGTTCCACTGAGCGTCAGACCCCGTAGAAAAGATC
AAAGGATCTTCTTGAGATCCTTTTTTTCTGCGCGTAATCTGCTGCTTGCAAACAAAAAAACCACCGCTACCAGCGGTGGTTTGTTTGCCG
GATCAAGAGCTACCAACTCTTTTTCCGAAGGTAACTGGCTTCAGCAGAGCGCAGATACCAAATACTGTCCTTCTAGTGTAGCCGTAGTTA
GGCCACCACTTCAAGAACTCTGTAGCACCGCCTACATACCTCGCTCTGCTAATCCTGTTACCAGTGGCTGCTGCCAGTGGCGATAAGTCG
TGTCTTACCGGGTTGGACTCAAGACGATAGTTACCGGATAAGGCGCAGCGGTCGGGCTGAACGGGGGGTTCGTGCACACAGCCCAGCTTG
GAGCGAACGACCTACACCGAACTGAGATACCTACAGCGTGAGCATTGAGAAAGCGCCACGCTTCCCGAAGGGAGAAAGGCGGACAGGTAT
CCGGTAAGCGGCAGGGTCGGAACAGGAGAGCGCACGAGGGAGCTTCCAGGGGGAAACGCCTGGTATCTTTATAGTCCTGTCGGGTTTCGC
CACCTCTGACTTGAGCGTCGATTTTTGTGATGCTCGTCAGGGGGGCGGAGCCTATGGAAAAACGCCAGCAACGCGGCCTTTTTACGGTTC
CTGGCCTTTTGCTGGCCTTTTGCTCACATGTT

SlmiR482b target site
***AtTAS1c*-derived spacer**
M13-F binding site
M13-Reverse binding site
attL1
attL2
Chloramphenicol resistance gene
*ccd*B gene
*Bsa*I site
inverted *Bsa*I site
Kanamycin resistance gene

>pMDC32B-SlmiR482bTS-B/c (11635 bp)

CCAGCCAGCCAACAGCTCCCCGACCGGCAGCTCGGCACAAAATCACCACTCGATACAGGCAGCCCATCAGTCCGGGACGGCGTCAGCGGG
AGAGCCGTTGTAAGGCGGCAGACTTTGCTCATGTTACCGATGCTATTCGGAAGAACGGCAACTAAGCTGCCGGGTTTGAAACACGGATGA
TCTCGCGGAGGGTAGCATGTTGATTGTAACGATGACAGAGCGTTGCTGCCTGTGATCACCGCGGTTTCAAAATCGGCTCCGTCGATACTA
TGTTATACGCCAACTTTGAAAACAACTTTGAAAAAGCTGTTTTCTGGTATTTAAGGTTTTAGAATGCAAGGAACAGTGAATTGGAGTTCG
TCTTGTTATAATTAGCTTCTTGGGGTATCTTTAAATACTGTAGAAAAGAGGAAGGAAATAATAAATGGCTAAAATGAGAATATCACCGGA
ATTGAAAAAACTGATCGAAAAATACCGCTGCGTAAAAGATACGGAAGGAATGTCTCCTGCTAAGGTATATAAGCTGGTGGGAGAAAATGA
AAACCTATATTTAAAAATGACGGACAGCCGGTATAAAGGGACCACCTATGATGTGGAACGGGAAAAGGACATGATGCTATGGCTGGAAGG
AAAGCTGCCTGTTCCAAAGGTCCTGCACTTTGAACGGCATGATGGCTGGAGCAATCTGCTCATGAGTGAGGCCGATGGCGTCCTTTGCTC
GGAAGAGTATGAAGATGAACAAAGCCCTGAAAAGATTATCGAGCTGTATGCGGAGTGCATCAGGCTCTTTCACTCCATCGACATATCGGA
TTGTCCCTATACGAATAGCTTAGACAGCCGCTTAGCCGAATTGGATTACTTACTGAATAACGATCTGGCCGATGTGGATTGCGAAAACTG
GGAAGAAGACACTCCATTTAAAGATCCGCGCGAGCTGTATGATTTTTTAAAGACGGAAAAGCCCGAAGAGGAACTTGTCTTTTCCCACGG
CGACCTGGGAGACAGCAACATCTTTGTGAAAGATGGCAAAGTAAGTGGCTTTATTGATCTTGGGAGAAGCGGCAGGGCGGACAAGTGGTA
TGACATTGCCTTCTGCGTCCGGTCGATCAGGGAGGATATCGGGGAAGAACAGTATGTCGAGCTATTTTTTGACTTACTGGGGATCAAGCC
TGATTGGGAGAAAATAAAATATTATATTTTACTGGATGAATTGTTTTAGTACCTAGAATGCATGACCAAAATCCCTTAACGTGAGTTTTC
GTTCCACTGAGCGTCAGACCCCGTAGAAAAGATCAAAGGATCTTCTTGAGATCCTTTTTTTCTGCGCGTAATCTGCTGCTTGCAAACAAA
AAAACCACCGCTACCAGCGGTGGTTTGTTTGCCGGATCAAGAGCTACCAACTCTTTTTCCGAAGGTAACTGGCTTCAGCAGAGCGCAGAT
ACCAAATACTGTCCTTCTAGTGTAGCCGTAGTTAGGCCACCACTTCAAGAACTCTGTAGCACCGCCTACATACCTCGCTCTGCTAATCCT
GTTACCAGTGGCTGCTGCCAGTGGCGATAAGTCGTGTCTTACCGGGTTGGACTCAAGACGATAGTTACCGGATAAGGCGCAGCGGTCGGG
CTGAACGGGGGGTTCGTGCACACAGCCCAGCTTGGAGCGAACGACCTACACCGAACTGAGATACCTACAGCGTGAGCTATGAGAAAGCGC
CACGCTTCCCGAAGGGAGAAAGGCGGACAGGTATCCGGTAAGCGGCAGGGTCGGAACAGGAGAGCGCACGAGGGAGCTTCCA GGGGGAAA
CGCCTGGTATCTTTATAGTCCTGTCGGGTTTCGCCACCTCTGACTTGAGCGTCGATTTTTGTGATGCTCGTCAGGGGGGCGGAGCCTATG
GAAAAACGCCAGCAACGCGGCCTTTTTACGGTTCCTGGCCTTTTGCTGGCCTTTTGCTCACATGTTCTTTCCTGCGTTATCCCCTGATTC
TGTGGATAACCGTATTACCGCCTTTGAGTGAGCTGATACCGCTCGCCGCAGCCGAACGACCGAGCGCAGCGAGTCAGTGAGCGAGGAAGC
GGAAGAGCGCCTGATGCGGTATTTTCTCCTTACGCATCTGTGCGGTATTTCACACCGCATATGGTGCACTCTCAGTACAATCTGCTCTGA
TGCCGCATAGTTAAGCCAGTATACACTCCGCTATCGCTACGTGACTGGGTCATGGCTGCGCCCCGACACCCGCCAACACCCGCTGACGCG
CCCTGACGGGCTTGTCTGCTCCCGGCATCCGCTTACAGACAAGCTGTGACCGTCTCCGGGAGCTGCATGTGTCAGAGGTTTTCACCGTCA
TCACCGAAACGCGCGAGGCAGGGTGCCTTGATGTGGGCGCCGGCGGTCGAGTGGCGACGGCGCGGCTTGTCCGCGCCCTGGTAGATTGCC
TGGCCGTAGGCCAGCCATTTTTGAGCGGCCAGCGGCCGCGATAGGCCGACGCGAAGCGGCGGGGCGTAGGGAGCGCAGCGACCGAAGGGT
AGGCGCTTTTTGCAGCTCTTCGGCTGTGCGCTGGCCAGACAGTTATGCACAGGCCAGGCGGGTTTTAAGAGTTTTAATAAGTTTTAAAGA
GTTTTAGGCGGAAAAATCGCCTTTTTTCTCTTTTATATCAGTCACTTACATGTGTGACCGGTTCCCAATGTACGGCTTTGGGTTCCCAAT
GTACGGGTTCCGGTTCCCAATGTACGGCTTTGGGTTCCCAATGTACGTGCTATCCACAGGAAAGAGAACTTTTCGACCTTTTTCCCCTGC
TAGGGCAATTTGCCCTAGCATCTGCTCCGTACATTAGGAACCGGCGGATGCTTCGCCCTCGATCAGGTTGCGGTAGCGCATGACTAGGAT
CGGGCCAGCCTGCCCCGCCTCCTCCTTCAAATCGTACTCCGGCAGGTCATTTGACCCGATCAGCTTGCGCACGGTGAAACAGAACTTCTT
GAACTCTCCGGCGCTGCCACTGCGTTCGTAGATCGTCTTGAACAACCATCTGGCTTCTGCCTTGCCTGCGGCGCGGCGTGCCAGGCGGTA
GAGAAAACGGCCGATGCCGGGATCGATCAAAAAGTAATCGGGGTGAACCGTCAGCACGTCCGGGTTCTTGCCTTCTGTGATCTCGCGGTA
CATCCAATCAGCTAGCTCGATCTCGATGTACTCCGGCCGCCCGGTTTCGCTCTTTACGATCTTGTAGCGGCTAATCAAGGCTTCACCCTC
GGATACCGTCACCAGGCGGCCGTTCTTGGCCTTCTTCGTACGCTGCATGGCAACGTGCGTGGTGTTTAACCGAATGCAGGTTTCTACCAG
GTCGTCTTTCTGCTTTCCGCCATCGGCTCGCCGGCAGAACTTGAGTACGTCCGCAACGTGTGGACGGAACACGCGGCCGGGCTTGTCTCC
CTTCCCTTCCCGGTATCGGTTCATGGATTCGGTTAGATGGGAAACCGCCATCAGTACCAGGTCGTAATCCCACACACTGGCCATGCCGGC
CGGCCCTGCGGAAACCTCTACGTGCCCGTCTGGAAGCTCGTAGCGGATCACCTCGCCAGCTCGTCGGTCACGCTTCGACAGACGGAAAAC
GGCCACGTCCATGATGCTGCGACTATCGCGGGTGCCCACGTCATAGAGCATCGGAACGAAAAAATCTGGTTGCTCGTCGCCCTTGGGCGG
CTTCCTAATCGACGGCGCACCGGCTGCCGGCGGTTGCCGGGATTCTTTGCGGATTCGATCAGCGGCCGCTTGCCACGATTCACCGGGGCG
TGCTTCTGCCTCGATGCGTTGCCGCTGGGCGGCCTGCGCGGCCTTCAACTTCTCCACCAGGTCATCACCCAGCGCCGCGCCGATTTGTAC
CGGGCCGGATGGTTTGCGACCGTCACGCCGATTCCTCGGGCTTGGGGGTTCCAGTGCCATTGCAGGGCCGGCAGACAACCCAGCCGCTTA
CGCCTGGCCAACCGCCCGTTCCTCCACACATGGGGCATTCCACGGCGTCGGTGCCTGGTTGTTCTTGATTTTCCATGCCGCCTCCTTTAG
CCGCTAAAATTCATCTACTCATTTATTCATTTGCTCATTTACTCTGGTAGCTGCGCGATGTATTCAGATAGCAGCTCGGTAATGGTCTTG
CCTTGGCGTACCGCGTACATCTTCAGCTTGGTGTGATCCTCCGCCGGCAACTGAAAGTTGACCCGCTTCATGGCTGGCGTGTCTGCCAGG
CTGGCCAACGTTGCAGCCTTGCTGCTGCGTGCGCTCGGACGGCCGGCACTTAGCGTGTTTGTGCTTTTGCTCATTTTCTCTTTACCTCAT
TAACTCAAATGAGTTTTGATTTAATTTCAGCGGCCAGCGCCTGGACCTCGCGGGCAGCGTCGCCCTCGGGTTCTGATTCAAGAACGGTTG
TGCCGGCGGCGGCAGTGCCTGGGTAGCTCACGCGCTGCGTGATACGGGACTCAAGAATGGGCAGCTCGTACCCGGCCAGCGCCTCGGCAA
CCTCACCGCCGATGCGCGTGCCTTTGATCGCCCGCGACACGACAAAGGCCGCTTGTAGCCTTCCATCCGTGACCTCAATGCGCTGCTTAA
CCAGCTCCACCAGGTCGGCGGTGGCCCATATGTCGTAAGGGCTTGGCTGCACCGGAATCAGCACGAAGTCGGCTGCCTTGATCGCGGACA
CAGCCAAGTCCGCCGCCTGGGGCGCTCCGTCGATCACTACGAAGTCGCGCCGGCCGATGGCCTTCACGTCGCGGTCAATCGTCGGGCGGT
CGATGCCGACAACGGTTAGCGGTTGATCTTCCCGCACGGCCGCCCAATCGCGGGCACTGCCCTGGGGATCGGAATCGACTAACAGAACAT
CGGCCCCGGCGAGTTGCAGGGCGCGGGCTAGATGGGTTGCGATGGTCGTCTTGCCTGACCCGCCTTTCTGGTTAAGTACAGCGATAACCT
TCATGCGTTCCCCTTGCGTATTTGTTTATTTACTCATCGCATCATATACGCAGCGACCGCATGACGCAAGCTGTTTTACTCAAATACACA
TCACCTTTTTAGACGGCGGCGCTCGGTTTCTTCAGCGGCCAAGCTGGCCGGCCAGGCCGCCAGCTTGGCATCAGACAAAC CGGCCAGGAT
TTCATGCAGCCGCACGGTTGAGACGTGCGCGGGCGGCTCGAACACGTACCCGGCCGCGATCATCTCCGCCTCGATCTCTTCGGTAATGAA
AAACGGTTCGTCCTGGCCGTCCTGGTGCGGTTTCATGCTTGTTCCTCTTGGCGTTCATTCTCGGCGGCCGCCAGGGCGTCGGCCTCGGTC
AATGCGTCCTCACGGAAGGCACCGCGCCGCCTGGCCTCGGTGGGCGTCACTTCCTCGCTGCGCTCAAGTGCGCGGTACAGGGTCGAGCGA
TGCACGCCAAGCAGTGCAGCCGCCTCTTTCACGGTGCGGCCTTCCTGGTCGATCAGCTCGCGGGCGTGCGCGATCTGTGCCGGGGTGAGG
GTAGGGCGGGGGCCAAACTTCACGCCTCGGGCCTTGGCGGCCTCGCGCCCGCTCCGGGTGCGGTCGATGATTAGGGAACGCTCGAACTCG

GCAATGCCGGCGAACACGGTCAACACCATGCGGCCGGCCGGCGTGGTGGTGTCGGCCCACGGCTCTGCCAGGCTACGCAGGCCCGCGCCG
GCCTCCTGGATGCGCTCGGCAATGTCCAGTAGGTCGCGGGTGCTGCGGGCCAGGCGGTCTAGCCTGGTCACTGTCACAACGTCGCCAGGG
CGTAGGTGGTCAAGCATCCTGGCCAGCTCCGGGCGGTCGCGCCTGGTGCCGGTGATCTTCTCGGAAAACAGCTTGGTGCAGCCGGCCGCG
TGCAGTTCGGCCCGTTGGTTGGTCAAGTCCTGGTCGTCGGTGCTGACGCGGGCATAGCCCAGCAGGCCAGCGGCGGCGCTCTTGTTCATG
GCGTAATGTCTCCGGTTCTAGTCGCAAGTATTCTACTTTATGCGACTAAAACACGCGACAAGAAAACGCCAGGAAAAGGGCAGGGCGGCA
GCCTGTCGCGTAACTTAGGACTTGTGCGACATGTCGTTTTCAGAAGACGGCTGCACTGAACGTCAGAAGCCGACTGCACTATAGCAGCGG
AGGGGTTGGATCAAAGTACTTTGATCCCGAGGGGAACCCTGTGGTTGGCATGCACATACAAATGGACGAACGGATAAACCTTTTCACGCC
CTTTTAAATATCCGTTATTCTAATAAACGCTCTTTTCTCTTAGGTTTACCCGCCAATATATCCTGTCAAACACTGATAGTTTAAACTGAA
GGCGGGAAACGACAATCTGATCCAAGCTCAAGCTGCTCTAGCATTCGCCATTCAGGCTGCGCAACTGTTGGGAAGGGCGATCGGTGCGGG
CCTCTTCGCTATTACGCCAGCTGGCGAAAGGGGGATGTGCTGCAAGGCGATTAAGTTGGGTAACGCCAGGGTTTTCCCAGTCACGACGTT
GTAAAACGACGGCCAGTGCCAAGCTTGGCGTGCCTGCAGGTCAACATGGTGGAGCACGACACACTTGTCTACTCCAAAAATATCAAAGAT
ACAGTCTCAGAAGACCAAAGGGCAATTGAGACTTTTCAACAAAGGGTAATATCCGGAAACCTCCTCGGATTCCATTGCCCAGCTATCTGT
CACTTTATTGTGAAGATAGTGGAAAAGGAAGGTGGCTCCTACAAATGCCATCATTGCGATAAAGGAAAGGCCATCGTTGAAGATGCCTCT
GCCGACAGTGGTCCCAAAGATGGACCCCCACCCACGAGGAGCATCGTGGAAAAAGAAGACGTTCCAACCACGTCTTCAAAGCAAGTGGAT
TGATGTGATAACATGGTGGAGCACGACACACTTGTCTACTCCAAAAATATCAAAGATACAGTCTCAGAAGACCAAAGGGCAATTGAGACT
TTTCAACAAAGGGTAATATCCGGAAACCTCCTCGGATTCCATTGCCCAGCTATCTGTCACTTTATTGTGAAGATAGTGGAAAAGGAAGGT
GGCTCCTACAAATGCCATCATTGCGATAAAGGAAAGGCCATCGTTGAAGATGCCTCTGCCGACAGTGGTCCCAAAGATGGACCCCCACCC
ACGAGGAGCATCGTGGAAAAAGAAGACGTTCCAACCACGTCTTCAAAGCAAGTGGATTGATGTGATATCTCCACTGACGTAAGGGATGAC
GCACAATCCCACTATCCTTCGCAAGACCCTTCCTCTATATAAGGAAGTTCATTTCATTTGGAGAGGACCTCGACTCTAGAGGATCCCCGG
GTACCGGGCCCCCCCTCGAGGCGCGCCAAGCTATCAAACAAGTTTGTACAAAAAAGCAGGCTCCGCGGCCGCCCCCTTCACCTGTA**GGCA**
TGGGCGGTGTAGGCAAGATAGACCATTTAAGAGACCATTAGGCACCCCAGGCTTTACACTTTATGCTTCCGGCTCGTATAATGTGTGGAT
TTTGAGTTAGGAGCCGTCGAGATTTTCAGGAGCTAAGGAAGCTAAAATGGAGAAAAAAATCACTGGATATACCACCGTTGATATATCCCA
ATGGCATCGTAAAGAACATTTTGAGGCATTTCAGTCAGTTGCTCAATGTACCTATAACCAGACCGTTCAGCTGGATATTACGGCCTTTTT
AAAGACCGTAAAGAAAAATAAGCACAAGTTTTATCCGGCCTTTATTCACATTCTTGCCCGCCTGATGAATGCTCATCCGGAGTTCCGTAT
GGCAATGAAAGACGGTGAGCTGGTGATATGGGATAGTGTTCACCCTTGTTACACCGTTTTCCATGAGCAAACTGAAACGTTTTCATCGCT
CTGGAGTGAATACCACGACGATTTCCGGCAGTTTCTACACATATATTCGCAAGATGTGGCGTGTTACGGTGAAAACCTGGCCTATTTCCC
TAAAGGGTTTATTGAGAATATGTTTTTCGTCTCAGCCAATCCCTGGGTGAGTTTCACCAGTTTTGATTTAAACGTGGCCAATATGGACAA
CTTCTTCGCCCCCGTTTTCACCATGGGCAAATATTATACGCAAGGCGACAAGGTGCTGATGCCGCTGGCGATTCAGGTTCATCATGCCGT
TTGTGATGGCTTCCATGTCGGCAGAATGCTTAATGAATTACAACAGTACTGCGATGAGTGGCAGGGCGGGGCGTAAACGCGTGGAGCCGG
CTTACTAAAAGCCAGATAACAGTATGCGTATTTGCGCGCTGATTTTTGCGGTATAAGAATATATACTGATATGTATACCCGAAGTATGTC
AAAAAGAGGTATGCTATGAAGCAGCGTATTACAGTGACAGTTGACAGCGACAGCTATCAGTTGCTCAAGGCATATATGATGTCAATATCT
CCGGTCTGGTAAGCACAACCATGCAGAATGAAGCCCGTCGTCTGCGTGCCGAACGCTGGAAAGCGGAAAATCAGGAAGGGATGGCTGAGG
TCGCCCGGTTTATTGAAATGAACGGCTCTTTTGCTGACGAGAACAGGGGCTGGTGAAATGCAGTTTAAGGTTTACACCTATAAAAGAGAG
AGCCGTTATCGTCTGTTTGTGGATGTACAGAGTGATATTATTGACACGCCCGGCCGACGGATGGTGATCCCCCTGGCCAGTGCACGTCTG
CTGTCAGATAAAGTCTCCCGTGAACTTTACCCGGTGGTGCATATCGGGGATGAAAGCTGGCGCATGATGACCACCGATATGGCCAGTGTG
CCGGTTTCCGTTATCGGGGAAGAAGTGGCTGATCTCAGCCACCGCGAAAATGACATCAAAAACGCCATTAACCTGATGTTCTGGGGAATA
TAAATGTCAGGCTCCCTTATACACAGCCAGTCTGCACCTCGACGGTCTCACATTAAGGGTGGGCGCGCCGACCCAGCTTTCTTGTACAAA
GTGGTTCGATAATTCCTTAATTAACTAGTTCTAGAGCGGCCGCCCACCGCGGTGGAGCTCGAATTTCCCCGATCGTTCAAACATTTGGCA
ATAAAGTTTCTTAAGATTGAATCCTGTTGCCGGTCTTGCGATGATTATCATATAATTTCTGTTGAATTACGTTAAGCATGTAATAATTAA
CATGTAATGCATGACGTTATTTATGAGATGGGTTTTTATGATTAGAGTCCCGCAATTATACATTTAATACGCGATAGAAAACAAAATATA
GCGCGCAAACTAGGATAAATTATCGCGCGCGGTGTCATCTATGTTACTGAATTCGTAATCATGGTCATAGCTGTTTCCTGTGTGAAATTG
TTATCCGCTCACAATTCCACACAACATACGAGCCGGAAGCATAAAGTGTAAAGCCTGGGGTGCCTAATGAGTGAGCTAACTCACATTAAT
TGCGTTGCGCTCACTGCCCGCTTTCCAGTCGGGAAACCTGTCGTGCCAGCTGCATTAATGAATCGGCCAACGCGCGGGGAGAGGCGGTTT
GCGTATTGGCTAGAGCAGCTTGCCAACATGGTGGAGCACGACACTCTCGTCTACTCCAAGAATATCAAAGATACAGTCTCAGAAGACCAA
AGGGCTATTGAGACTTTTCAACAAAGGGTAATATCGGGAAACCTCCTCGGATTCCATTGCCCAGCTATCTGTCACTTCATCAAAAGGACA
GTAGAAAAGGAAGGTGGCACCTACAAATGCCATCATTGCGATAAAGGAAAGGCTATCGTTCAAGATGCCTCTGCCGACAGTGGTCCCAAA
GATGGACCCCCACCCACGAGGAGCATCGTGGAAAAAGAAGACGTTCCAACCACGTCTTCAAAGCAAGTGGATTGATGTGATAACATGGTG
GAGCACGACACTCTCGTCTACTCCAAGAATATCAAAGATACAGTCTCAGAAGACCAAAGGGCTATTGAGACTTTTCAACAAAGGGTAATA
TCGGGAAACCTCCTCGGATTCCATTGCCCAGCTATCTGTCACTTCATCAAAAGGACAGTAGAAAAGGAAGGTGGCACCTACAAATGCCAT
CATTGCGATAAAGGAAAGGCTATCGTTCAAGATGCCTCTGCCGACAGTGGTCCCAAAGATGGACCCCCACCCACGAGGAGCATCGTGGAA
AAAGAAGACGTTCCAACCACGTCTTCAAAGCAAGTGGATTGATGTGATATCTCCACTGACGTAAGGGATGACGCACAATCCCACTATCCT
TCGCAAGACCTTCCTCTATATAAGGAAGTTCATTTCATTTGGAGAGGACACGCTGAAATCACCAGTCTCTCTCTACAAATCTATCTCTCT
CGAGCTTTCGCAGATCCCGGGGGGCAATGAGATATGAAAAAGCCTGAACTCACCGCGACGTCTGTCGAGAAGTTTCTGATCGAAAAGTTC
GACAGCGTCTCCGACCTGATGCAGCTCTCGGAGGGCGAAGAATCTCGTGCTTTCAGCTTCGATGTAGGAGGGCGTGGATATGTCCTGCGG
GTAAATAGCTGCGCCGATGGTTTCTACAAAGATCGTTATGTTTATCGGCACTTTGCATCGGCCGCGCTCCCGATTCCGGAAGTGCTTGAC
ATTGGGGAGTTTAGCGAGAGCCTGACCTATTGCATCTCCCGCCGTGCACAGGGTGTCACGTTGCAAGACCTGCCTGAAACCGAACTGCCC
GCTGTTCTACAACCGGTCGCGGAGGCTATGGATGCGATCGCTGCGGCCGATCTTAGCCAGACGAGCGGGTTCGGCCCATTCGGACCGCAA
GGAATCGGTCAATACACTACATGGCGTGATTTCATATGCGCGATTGCTGATCCCCATGTGTATCACTGGCAAACTGTGATGGACGACACC
GTCAGTGCGTCCGTCGCGCAGGCTCTCGATGAGCTGATGCTTTGGGCCGAGGACTGCCCCGAAGTCCGGCACCTCGTGCACGCGGATTTC
GGCTCCAACAATGTCCTGACGGACAATGGCCGCATAACAGCGGTCATTGACTGGAGCGAGGCGATGTTCGGGGATTCCCAATACGAGGTC
GCCAACATCTTCTTCTGGAGGCCGTGGTTGGCTTGTATGGAGCAGCAGACGCGCTACTTCGAGCGGAGGCATCCGGAGCTTGCAGGATCG
CCACGACTCCGGGCGTATATGCTCCGCATTGGTCTTGACCAACTCTATCAGAGCTTGGTTGACGGCAATTTCGATGATGCAGCTTGGGCG
CAGGGTCGATGCGACGCAATCGTCCGATCCGGAGCCGGGACTGTCGGGCGTACACAAATCGCCCGCAGAAGCGCGGCCGTCTGGACCGAT

GGCTGTGTAGAAGTACTCGCCGATAGTGGAAACCGACGCCCCAGCACTCGTCCGAGGGCAAAGAAATAGAGTAGATGCCGACCGGATCTG
TCGATCGACAAGCTCGAGTTTCTCCATAATAATGTGTGAGTAGTTCCCAGATAAGGGAATTAGGGTTCCTATAGGGTTTCGCTCATGTGT
TGAGCATATAAGAAACCCTTAGTATGTATTTGTATTTGTAAAATACTTCTATCAATAAAATTTCTAATTCCTAAAACCAAAATCCAGTAC
TAAAATCCAGATCCCCCGAATTAATTCGGCGTTAATTCAGTACATTAAAAACGTCCGCAATGTGTTATTAAGTTGTCTAAGCGTCAATTT
GTTTACACCACAATATATCCTGCCA

SlmiR482b target site
***AtTAS1c*-derived spacer**
T-DNA right border
T-DNA left border
*ccd*B gene
*Bsa*I site
Inverted *Bsa*I site
Chloramphenicol resistance gene
attB1
attB2
Nos terminator
CaMV promoter
kanamycin resistance gene
Hygromycin resistance gene
2x35S CaMV promoter
CaMV terminator
